# Patterns and unique features of infantile cholestasis among Arabs

**DOI:** 10.3389/fped.2024.1423657

**Published:** 2024-07-30

**Authors:** Abdulrahman Al-Hussaini, Sami Alrashidi, Deema H. Hafez, Yasir S. Alkhalifah, Bashaer Otayn, Majid Alrasheed, Sumayah Al Mufarreh, Sultan AlKasim

**Affiliations:** ^1^ Division of Pediatric Gastroenterology, Children’s Specialized Hospital, King Fahad Medical City, Riyadh, Saudi Arabia; ^2^ College of Medicine, Alfaisal University, Riyadh, Saudi Arabia; ^3^ Prince Abdullah bin Khalid Celiac Disease Research Chair, Department of Pediatrics, Faculty of Medicine, King Saud University, Riyadh, Saudi Arabia; ^4^ Department of Pediatrics, Children’s Specialized Hospital, King Fahad Medical City, Riyadh, Saudi Arabia; ^5^ Department of Pediatrics, College of Medicine, Qassim University, Buraydah, Saudi Arabia; ^6^ Intensive Care Department, King Fahad Medical City, Riyadh, Saudi Arabia; ^7^ Pediatric Endocrinology Department, King Fahad Medical City, Riyadh, Saudi Arabia; ^8^ Department of Pediatrics, Prince Sultan Military Medical City, Riyadh, Saudi Arabia; ^9^ College of Medicine, King Saud University, Riyadh, Saudi Arabia

**Keywords:** liver disease, next-generation sequence, Saudi Arabia, whole exome sequence, idiopathic neonatal hepatitis

## Abstract

**Background:**

Most of the literature on infantile cholestasis (IC) originated from Caucasian and Asian populations. The differential diagnosis of IC is very broad, and identification of etiology is challenging to clinicians because the list includes many entities with overlapping clinical, biochemical, and histological features. Thus, a structured, stepwise diagnostic approach is required to help early recognition and prompt evaluation and management of treatable causes of cholestasis.

**Objective:**

(1) To determine the differential diagnosis of IC among Saudi population and (2) to evaluate the usefulness of a diagnostic algorithm that has been tailored by the authors to the local practice.

**Methods:**

All infants with onset of cholestasis before 12 months of age (2007 and 2020) were identified and included if they underwent extensive work up to exclude infectious, structural, metabolic, endocrine, infiltrative, and familial causes.

**Results:**

Our diagnostic pathway allowed a definite diagnosis in 373 of the included 533 cases; 160 (30%) were labelled as “idiopathic neonatal hepatitis” (INH) [i.e., overall 70% detection rate]. However, when considering the cases that underwent extensive investigations including advanced gene testing (415 of the 533), the yield of the diagnostic algorithm was 90% (373/415). Familial cholestasis group was the most common in 20% (107/533), and biliary atresia and neonatal-onset Dubin Johnson syndrome contributed to 6% each. The genetic/hereditary causes of cholestasis contributed to 58% of the diagnosed cases (217/373). No single case of alpha-1 antitrypsin deficiency was diagnosed. Forty-nine infants with cholestasis presented with liver failure (9%).

**Conclusion:**

Our study highlights several unique features and causes of IC among Arabs which could have a great impact on the differential diagnosis process and the choice of laboratory tests used in the clinical setting.

## Introduction

Infantile cholestasis (IC) is a common manifestation of several insults to the highly vulnerable liver in young infants with their limited capacity of bile acids synthesis and transport. The differential diagnosis of IC is very broad, and identification of etiology is challenging to clinicians because the list includes many entities with overlapping clinical, biochemical, and histological features. Thus, a structured, stepwise diagnostic approach is required that should incorporate clinical assessment, biochemical, radiological, histological, and/or molecular testing, to help early recognition and prompt evaluation and management of treatable causes of cholestasis. Notably, the recent advancement in molecular and biochemical technologies and their addition to the armamentarium used in the work up of IC have helped decoding the puzzle of idiopathic neonatal hepatitis (INH), a diagnosis of exclusion, by identifying several hereditary and metabolic liver diseases.

According to a meta-analysis, published in 2015 and analyzed data of 1,692 cases of IC from 17 studies, biliary atresia (BA) was the most common cause worldwide (26%); other etiologies comprised infection (12%), total parenteral nutrition (6%), metabolic disease (4.3%), alpha-1-antitrypsin deficiency (4.1%), and perinatal hypoxia/ischemia (3.6%) (1). Much of the literature to date originated from Caucasian and Asian populations, thus the epidemiology and pattern of IC remain unclear among Arabs. This missing information would have a great impact on the diagnostic algorithm used and the choice of laboratory tests in real clinical practice.

The objectives of our study were to determine the differential diagnosis and characterize the epidemiology and geographical distribution of IC among Saudi population, and to evaluate the usefulness of a diagnostic algorithm that has been tailored by the authors to the local practice.

## Patients and methods

### Study setting and design

King Fahad Medical City is one of the largest tertiary referral centers for children with liver disorders referred from all the 13 provinces in Saudi Arabia. The total population in Saudi Arabia is 35 million people; Riyadh region is the most populated with 8.5 million inhabitants. We retrospectively reviewed our database of IC that presented to our center in Riyadh city, the capital of Saudi Arabia, during the period from 2007 until 2020.

### Study population

All infants with onset of cholestasis before 12 months of age were identified and included if they met the following entry criteria: (1) cholestasis, defined clinically as presence of jaundice and/or acholic stools, with a conjugated bilirubin of >20 µmol/L, or itching in infants older than 6 months with total serum bile acids (TBA) >10 (Normal, 0–10 µmol/L); (2) underwent extensive work up to exclude infectious, structural, metabolic, endocrine, infiltrative, and familial causes. We excluded cases with undetermined etiology because of inadequate investigations, as outlined in level 1 of assessment below.

### Study procedures

#### Hospital diagnostic algorithm on approaching infants with cholestasis

**Figure 1:**
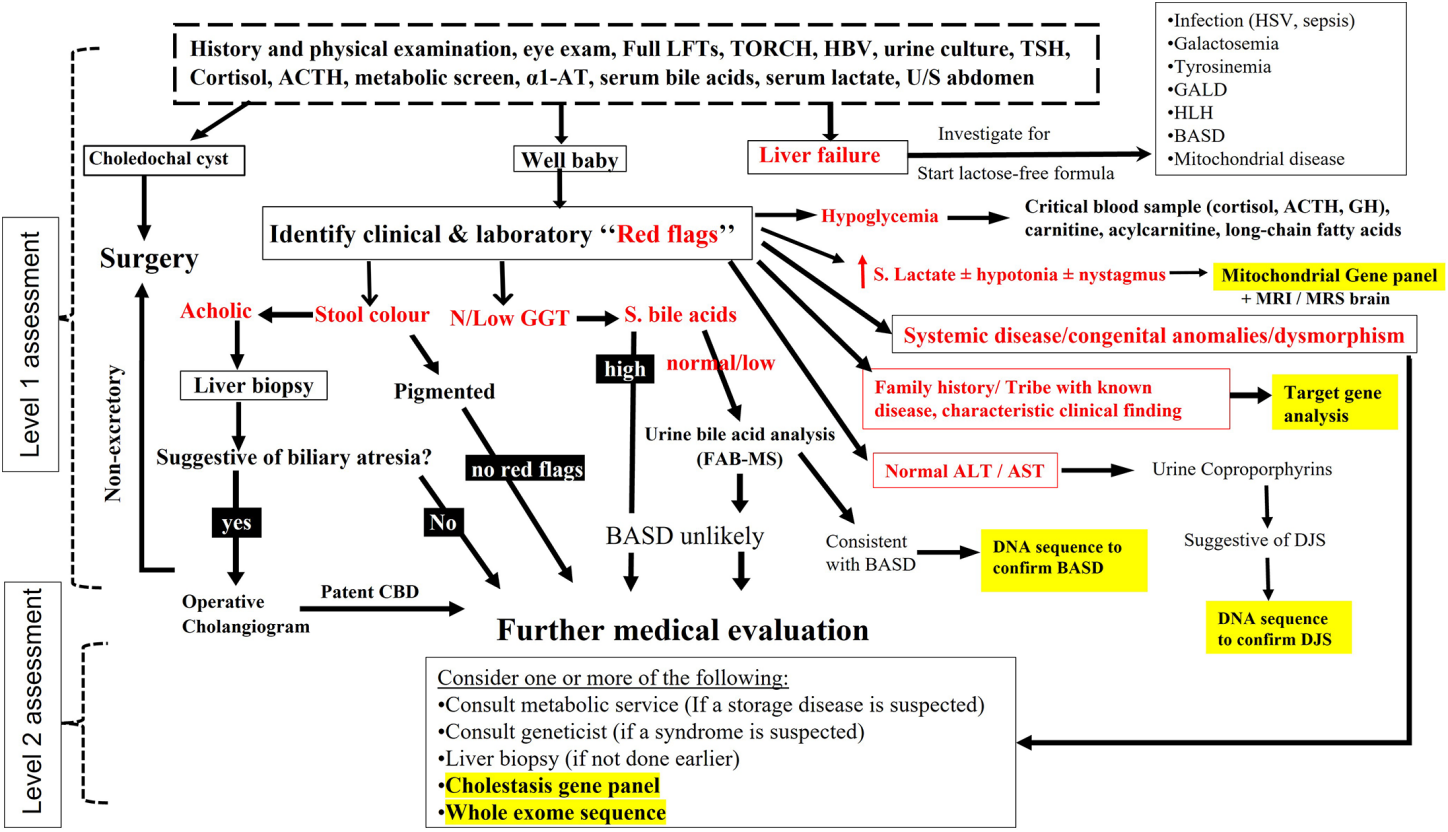
Schematic representation of the diagnostic protocol for infantile cholestasis adopted in our center. The “red flags” are written in bolded red color. The places in the algorithm where we have incorporated different types of gene tests are highlighted by yellow color.

All infants transferred to our center with cholestasis underwent a diagnostic algorithm depicted in Figure 1, which comprised two levels of assessment:

(1) Level 1 assessment included detailed history, physical examination, baseline laboratory, serological, and extensive biochemical investigations to diagnose underlying infectious, endocrine, and metabolic causes, and ultrasound abdomen (±magnetic resonance cholangiography) to diagnose biliary anomalies. In the first level of assessment, we have adopted a stepwise approach with high priority to promptly diagnose treatable disorders. In our protocol, we considered liver biopsy (LB) when BA was highly suspected [high gamma-glutamyl transferase (GGT) cholestasis, pale stool, and/or findings such as abdominal heterotaxy and splenic malformation]. During this stage of work up, we have looked carefully for clinical and laboratory “red flags” that helped focusing our investigations toward specific etiologies. Among the important clinical clues are similar family history of liver disease, consanguinity, the tribe, the region of origin, dysmorphic face, clinical findings that suggest a specific cause of cholestasis (e.g., arthrogryposis in ARC syndrome), and eye findings (e.g., cataract, cherry red spot). Among the laboratory tests, GGT had been used to stratify patients into high and low or normal level cholestasis; GGT is generally elevated in cholestasis except in few conditions such as bile acid synthesis disorder (BASD) and progressive familial intrahepatic cholestasis (PFIC) type 1 & 2. In our screening protocol for BASD, the presence of normal/low serum total bile acid (TBA) level (normal, 0–10 µmol/L) in a baby with normal GGT concentration has been an indication to send urine for mass-spectrometry to analyze for the atypical bile acid signature metabolites consistent with BASD, as we detailed elsewhere (2). Another helpful biochemical marker is serum lactate, which when persistently elevated (>2.2 mmol/L) in a baby with hypotonia (±nystagmus) has been an indication to investigate for mitochondrial hepatopathy, including mitochondrial gene panel, as we described previously (3). Sick young infant with IC and coagulopathy (Vitamin K non-responsive INR ≥ 2) prompted investigations for a list of specific diagnoses posted in Figure 1. Development of hypoglycemia in an infant with cholestasis and preserved liver synthetic function is an unusual event that we frequently noticed in endocrine and metabolic/mitochondrial disorders (3, 4). Elevated liver transaminases are markers of hepatocytes injury, which is a feature in the vast majority of causes of IC, however in hereditary conjugated bilirubin metabolism disorders [e.g., Dubin-Johnson syndrome (DJS) and Rotor syndrome], because there is no hepatocyte injury, the resulting direct hyperbilirubinemia is typically accompanied with normal alanine transaminase/aspartate transaminase (ALT/AST) levels (5). This important biochemical finding has shifted our diagnostic approach to request urine for coproporphyrins analysis to support the diagnosis. During “level 1” of work up and on high suspicion of a specific hereditary etiology (based on specific biochemical/metabolic test, positive family history, characteristic clinical findings e.g Alagille or ARC syndrome), we proceeded directly to confirmation by target gene analysis. When there was an abnormal complete blood counts, we consulted hematologic services to investigate for infiltrative diseases (bone marrow biopsy). Presence of clinical findings consistent with a specific chromosomal disorder prompted use of fluorescence *in situ* hybridization (FISH) to confirm the diagnosis.
(2) Level 2 of assessment: when the diagnosis remained undetermined after the extensive investigations in level 1, we initiated level 2 of work up which included several investigations as dictated by the clinical assessment (Figure 1). During the first half of the study period, and before availability of advanced molecular testing to us, we frequently considered percutaneous LB at this stage in selected cases if it was not performed previously for cases suspicious of BA. When there was marked splenomegaly, we consulted metabolic service to investigate for storage diseases (measure lysosomal enzyme activities in blood or cultured skin fibroblast, and/or lysosomal storage disorders panel. All infants with cholestasis with no clear clues to a definite etiology underwent molecular analysis using a cholestasis panel (Supplementary Table S1). In case the panel did not reveal any molecular diagnosis, the analysis was extended to whole exome sequence (WES). If the cholestasis resolved but the diagnosis remained undetermined, a cholestasis panel was offered only in selected cases in whom family history was strongly suggestive of a genetic condition (e.g., affected siblings or relatives). WES was performed as the first gene testing in patients with complex phenotypes (systemic involvement, congenital anomalies, non-specific dysmorphic features, and/or developmental delay) but no clear clues to a definite etiology, after consulting the genetic service.

All infants with cholestasis received supplementation with fat-soluble vitamins and ursodeoxycholic acid therapy. Infants presenting with liver failure were initiated on lactose free formula until galactosemia was ruled out.

#### Data collection

Medical records were reviewed for final diagnosis, demographics, gestational age, age at onset of jaundice, age at referral, family history, and consanguinity.

#### Molecular genetic investigations

Our selection of genetic tests evolved since 2007 as was the progress of genetic analysis technologies over the past 2 decades. Initially, we used direct sequencing for selected genes based on the phenotype of a patient. This was followed by use of “Jaundice chip” that sequence a limited number of genes (SERPINA1, JAG1, ATP8B1, ABCB11, and ABCB4) (6). Since 2012, we have used next generation sequencing (NGS) panels in liver diseases that incorporated a larger number of genes beyond PFIC1-3 (Supplementary Table S1). The potential pathogenic variants were evaluated by the prediction programs, Mutation Taster, PolyPhen-2, and SIFT. For novel gene variants, we collected blood DNA samples from parents of patients were analyzed for the novel allele variants by Sanger sequencing, whenever possible. All the procedures were conducted with informed consent.

#### Definitions and diagnostic criteria

An infant was considered to have INH after the extensive evaluation in “level 1 work up” failed to identify the underlying cause of cholestasis and/or negative molecular analysis (gene panel or WES). In this study, the diagnoses of all hereditary diseases were confirmed by molecular analysis. All infants with inborn errors of metabolism and characteristic pattern of metabolites on metabolic screening tests were confirmed by molecular genetic diagnosis. Diagnosis of other etiologies (e.g endocrine, infectious, infiltrative) was based on disease-specific laboratory assays and criteria confirmed by the concerned pediatric specialty. The presence of a consistent liver histology (ductular proliferation, portal fibrosis, ductular cholestasis with bile plugs) and non-excretory cholangiography confirmed the diagnosis of BA. Diagnosis of gestational alloimmune liver disease (GALD) was based on demonstration of iron deposition in salivary glands on lip biopsy.

#### Ethical consideration

A written informed consent was obtained from parents before genetic testing. The study has been approved by the ethical committee of our hospital (number 14-009).

## Results

### The demographic and clinical characteristics of the infantile cholestasis cases

Five hundred fifty-three cases of IC presented to our center during the study period; 20 cases were excluded because of inadequate investigations. The remaining 533 cases underwent extensive investigations to warrant inclusion in the study (336 males; male: female ratio 1.58:1; 86% were full term and 14% pre-term). The onset of jaundice was at a mean age of 2 weeks ± 1.7 weeks and the mean age at presentation to our center was 10 weeks ± 8.5 weeks. Out of 533, 141 (26.5%) had pale stool at presentation and the remaining (73.5%) had pigmented stool. Consanguinity was present in 64% and a history of liver disease in first-degree relatives was documented in 23.5%.

### The differential diagnosis and etiologic categories of infantile cholestasis

The etiologies of IC, defined categorically by disease process, are outlined in Table 1. Idiopathic group accounted for the majority, occurring in 160 cases (30%), followed by the familial cholestasis group (20%), biliary/anatomic group (18.5%), and infectious causes (11%). The genetic/hereditary causes of cholestasis contributed to 58% of the diagnosed cases (217/373), 10 cases (3%) were due to chromosomal disorders, and the remaining 146 of the 373 cases were due to non-genetic causes (37%). The specific etiologies within each category are shown in Table 1. The most common underlying individual etiology was BA (*n* = 35; 6.5%) followed by PFIC 2 and neonatal onset-DJS (6%, each), Cytomegalovirus infection (5%), and Alagille syndrome and urinary tract infection (3.2%, each). Forty-nine infants with cholestasis presented with liver failure (9%). The causes of liver failure in infancy are listed in Table 2.

**Table 1:** Categories and etiologies of infantile cholestasis in Saudi Arabia (*n *= 533 cases).

Categories/etiologies	No. (%)
Biliary/anatomic (*n* = 99, 18.6%)	
– Biliary atresia	35
– Choledochal cyst	6
– Bile duct paucity	
• Alagille syndrome	17
• William syndrome	1
• FOXA1 gene mutation	1
– Cystic fibrosis	8
– Inspissated bile	8
– Spontaneous perforation of CBD	2
– Ciliopathies	
• Caroli disease and congenital hepatic fibrosis	6
• Joubert syndrome	1
• Meckel gruber syndrome	1
• *TTC26* gene mutation	4
• *WDR19* gene mutation	1
• *WDR35* gene mutation	3
• *KIF 12* gene mutation	2
• *NPHP3* gene mutation	2
Familial cholestasis (*n* = 107, 20%)	
(A) Bile acid transport defects	
– *ATB8B1* gene mutations	5
– *ABCB11* gene mutations	32
– *ABCB4* gene mutations	1
– Dubin–Johnson syndrome	31
– Tight junction protein defects	
• *TJP2* gene mutation	6
• *USP53* gene mutation	2
• CLDN1 *gene mutation*	1
*– MYO5B* gene mutation	3
*– NR1H4* gene mutation	1
(B) Bile acid synthesis/metabolism defects	
– Primary bile acid synthesis disorders	
Δ^4^-3oxosteroid-5β-reductase deficiency (*HSD3B7*)	7
3β-hydroxy-Δ5-C27-steroiddehydrogenase def (*AKR1D1*)	8
– Primary bile acid malabsorption (*SLC10A2* gene mutation)	2
– Zellweger's syndrome *(PEX6* gene mutation)	2
– Cerebrotendinous Xanthomatosis—27-hydroxylase def *(CYP27A1)*	1
(C) Other cholestatic syndromes	
– ARC syndrome	2
*– FOCAD* gene mutation	1
– Lipolysis-stimulated lipoprotein receptor (*LSR* gene mutation)	1
*– IARS1* gene mutation	1
Metabolic/storage disease (*n* = 27, 5%)	
– Galactosemia	13
– Tyrosinemia	6
*– ACADVL* mutation (very long chain Acyl-CoA dehydrogenase Def)	1
– Gaucher's disease	2
– Niemann-Pick disease	5
Endocrine (*n* = 14, 2.6%)	
– Primary adrenal insufficiency (*MCT2R* gene mutation)	1
– ACTH deficiency	10
– Pan-hypopituitarism	3
Mitochondrial hepatopathy (*n* = 29, 5.5%)	
– MPV17 gene mutation	12
– DGOUK gene mutation	13
*– TRMU* gene mutation	1
– *SERAC1* gene mutation	2
– *FARS2* gene mutation	1
Immune-mediated (*n* = 5, 1%)	
– Gestational alloimmune liver disease	5
Infiltrative (*n* = 10, 2%)	
– Leukemia	3
– Hemophagocytic Lymphohistiocytosis (*STXBP2* gene mutation)	7
Infections (*n* = 59, 11%)	
– CMV	27
– HBV	2
– UTI	17
– Sepsis	13
Toxic (*n* = 9, 1.7%)	
– TPN	9
Miscellaneous (*n* = 14, 2.6%)	
– Down syndrome	7
– Edward syndrome	2
– McCune-Albright syndrome	1
– Immunodeficiency disorders	
• Omenn syndrome	1
• Common variable immunodeficiency	1
– Transaldolase deficiency	2
Idiopathic (*n* = 160, 30%)	

**Table 2:** Causes of infantile liver failure.

Cause	*N* = 49
Metabolic liver disease	
– Galactosemia	7 (14.4%)
– Tyrosinemia	6 (12.5%)
Hemophagocytic lymphohistiocytosis	6 (12.5%)
Gestational alloimmune liver disease	5 (10%)
Mitochondrial hepatopathy	3 (6%)
Miscellaneous	
– Transaldolase deficiency	2 (4%)
– Sepsis	2 (4%)
– Niemann-Pick disease	1 (2%)
– Bile acid synthesis defect	1 (2%)
– UPS53 Gene mutation	1 (2%)
– Congenital leukemia	1 (2%)
Idiopathic	14 (28.5%)

### The geographical distribution of the 533 cases of infantile cholestasis

**Figure 2:**
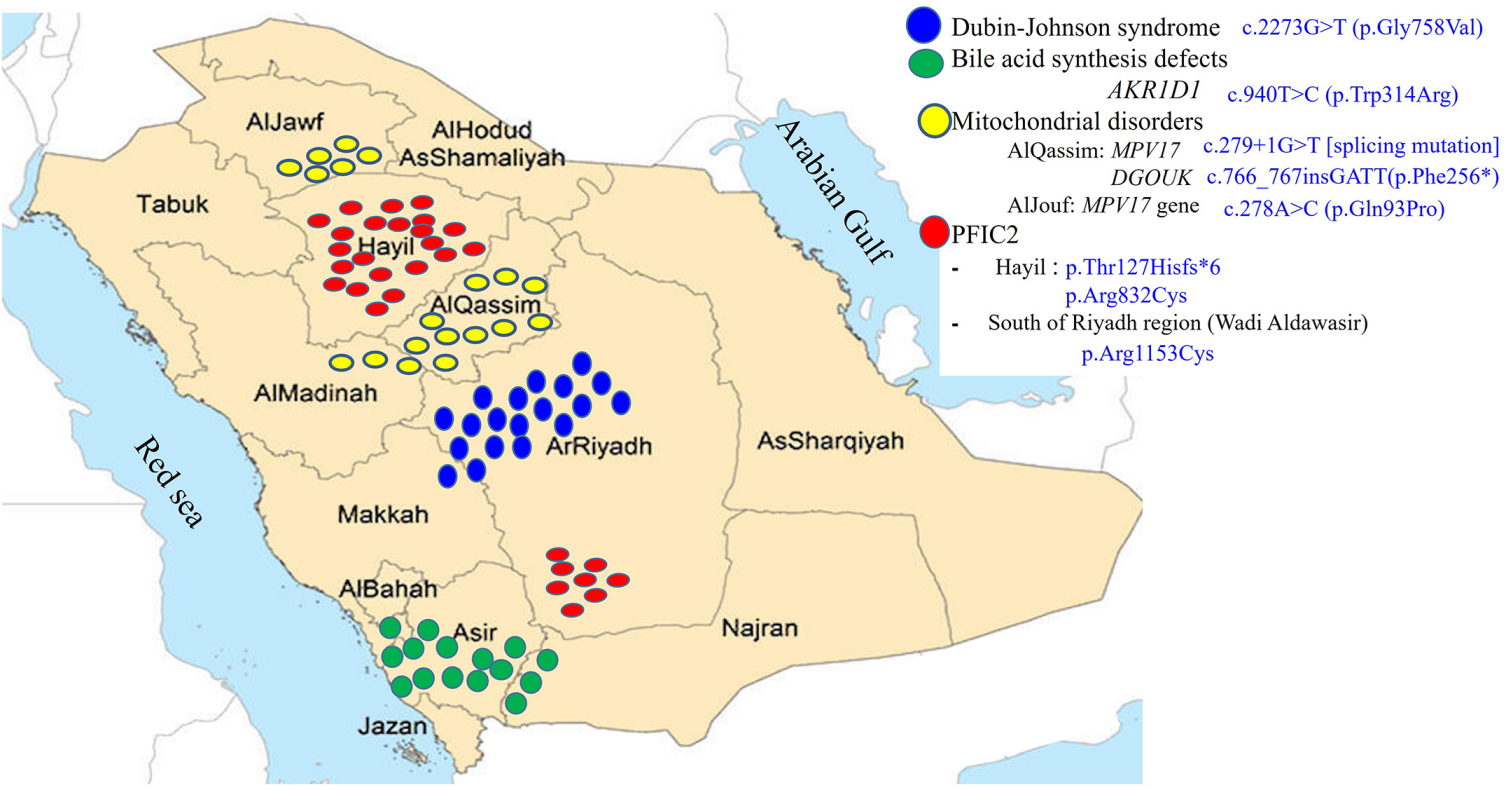
Regional distribution of the founder variants.

Forty five percent of the referrals were from Riyadh region and 55% were referred from the remaining 12 regions in Saudi Arabia with different percentages ranging from 2 to 7%. There is conspicuous clustering of cases due to 4 main hereditary causes of IC in certain regions of Saudi Arabia (Figure 2). Two thirds of the patients with PFIC2 (21 of 32, from 15 unrelated families) belong to two main tribes in Hail region; they harbor 2 pathologic variants, p.Thr127Hisfs*6 [frameshift mutation] and p.Arg832Cys [missense mutation]. There is a smaller focus of patients with PFIC2 in the South of Riyadh region in Aldawaser valley that carry p.Arg1153Cys [missense mutation]. Two thirds of the patients with DJS (19 of 31, from 16 unrelated families) belong to 5 major tribes in Riyadh region extending to Taif city in Makkah region. They harbor a homozygous missense mutation, p.Gly758Val. There is a noticeable cluster of cases with mitochondrial depletion syndrome in AlQassim region in a major tribe that extend to AlMadinah region; this tribe harbors c.279 + 1G > T [splicing mutation] in the *MPV17* gene and c.766_767insGATT(p.Phe256*) [insertion mutation] in the *DGUOK* gene. Another smaller focus of MPV17 cases is present in AlJouf region where 5 unrelated families from one major tribe harbor c.278A > C (p.Gln93Pro) [missense mutation] in the *MPV17* gene. Lastly, a prominent cluster of primary BASD was observed in the South-Western part of Saudi Arabia where 13 of the 15 BASD cases (7 un-related families from 3 tribes) reside.

### Evaluation of the diagnostic yield of the proposed pathway

**Figure 3:**
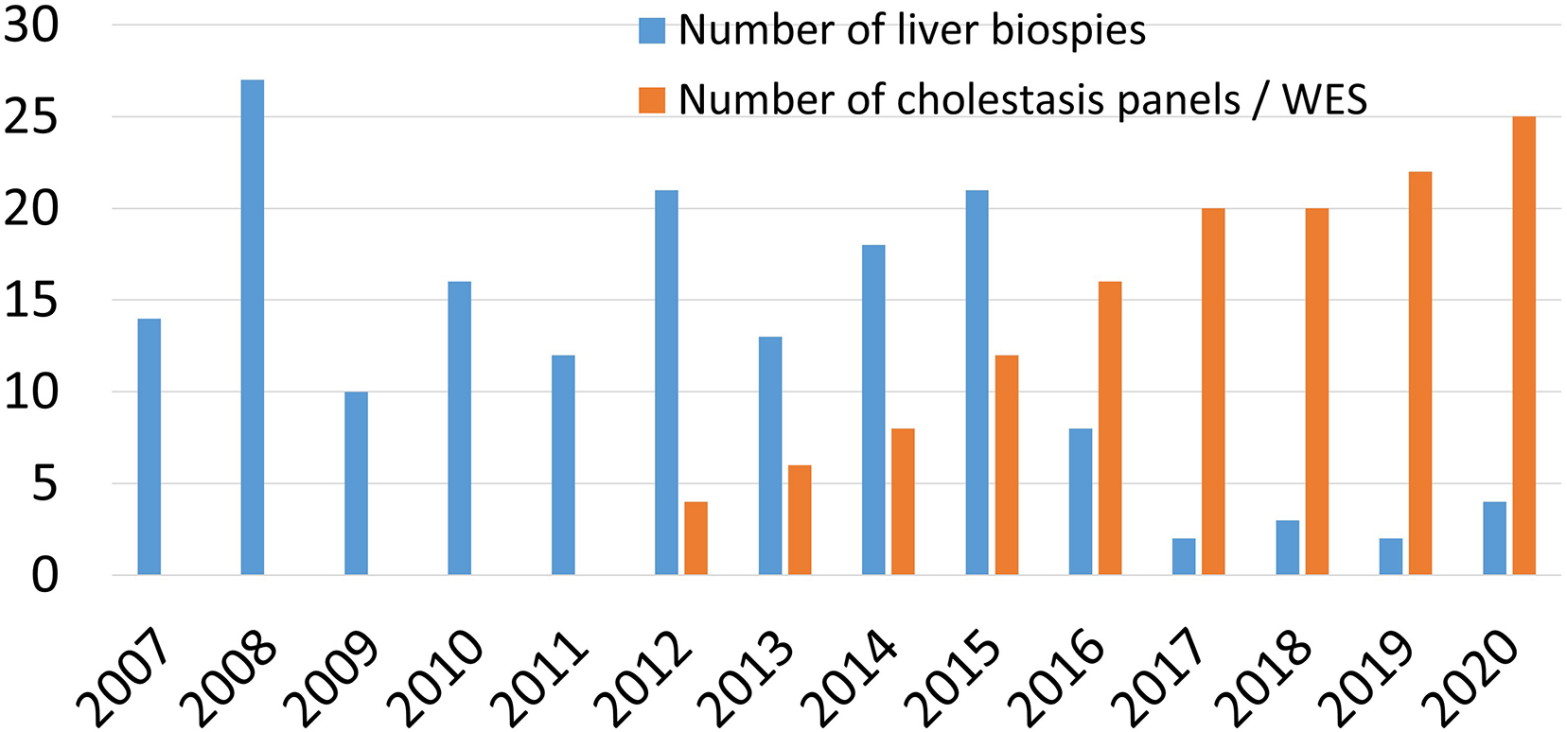
Time trend of the use of liver biopsy and gene testing in the work up of infantile cholestasis.

In the era prior to 2012 (2007–2011), the diagnostic yield of the diagnostic algorithm was 55% (110 cases were diagnosed out of 200) and rose to 90% beyond 2012 (after introduction of more advanced molecular testing). Overall, our diagnostic pathway allowed a definite diagnosis in 373 of the 533 cases, with a 70% detection rate. Two-hundred eighty-two cases (53%) were identified during the first level of assessment, including 146 cases with non-genetic cholestasis (27.4%), 10 cases with chromosomal disorder (Down syndrome in 7, Edward syndrome in 2, and William syndrome in one), and 126 cases of genetic cholestasis (23.6%). A total of 122 LBs were obtained (23%). Suspicion of BA was the most common indication for LB in 46 cases (37.8%). Figure 3 shows the time trend of use of LB and advanced molecular testing as part of our diagnostic work up of IC during the study period. The genetic causes of cholestasis diagnosed during level 1 of assessment included: Alagille syndrome (*n* = 14), CF (*n* = 8), PFIC2 (*n* = 8), DJS (*n* = 31), *TJP2* gene mutation (*n* = 1), *MYO5B* gene mutation (*n* = 3), BASD (*n* = 17), ARC syndrome (*n* = 2), mitochondrial hepatopathy (*n* = 15), galactosemia (*n* = 13), tyrosinemia (*n* = 6), primary adrenal insufficiency due to *MCT2R* gene mutation (*n* = 1), and familial HLH (*n* = 7).

Two-hundred fifty-one of the total 533 cases with IC remained without diagnosis after level 1 assessment. One hundred thirty-three of the 251 infants received advanced genetic testing using expanded cholestasis panel and/or WES; 91 of the 133 infants were confirmed to have a molecular diagnosis (68.5%) and the remaining 42 cases remained idiopathic (31.5%). Of the 133 patients, 70 received panel alone with 72% diagnostic yield]. WES was performed in 53 patients and confirmed the diagnosis in 65%. The remaining 10 patients tested negative for cholestasis panel and subsequently underwent WES (tested positive in 7). The remaining 118 infants with indeterminate etiology, although received extensive investigation (19 had target gene test specific for PFIC 1–3, prior to 2012; mitochondrial and storage disease panel in 58), however they did not undergo advanced molecular testing. Hence, of the total 533 cases with IC included in our study, 415 cases were extensively investigated, including advanced molecular testing; 373 of the 415 cases were diagnosed, and only 42 patients (10%) remained with indeterminate etiology, resulting in a diagnostic yield of 90%.

## Discussion

This is the largest case series reported to date worldwide that describes the clinical patterns and spectrum of causes of IC, and is the first among Arabs to provide a detailed updated analysis of IC in the era of advanced molecular and biochemical testing. Our study highlights several important unique features of IC in Saudi Arabia. First, the frequency of BA in our large study cohort is only 6.5% as compared to 20% to 42% reported in all the case series of IC reported from different populations (1). Consistent with this observation, we showed, in a multicenter national study, that the incidence of BA in Saudi Araba is 1 in 44,000 live births (7) which is much lower than reported by other national registries in East Asia (1: 2,700 to 1: 9,400 live births) and Europe (1: 12,000 to 19,000 live births) (8, 9). Second, the genetic/hereditary causes of IC contributed to 58% of the diagnosed cases (217/373) in contrast to 22% to 27% in cohorts of IC from North America and East Asia (10–13). The Saudi population is a tribal community with a high prevalence of consanguineous marriage (up to 57%) which presents a major risk factor for autosomal recessive diseases (14). The rate of consanguinity among our study cohort was 64%. As a result, the familial cholestasis category predominated contributing to overall 20% of the infants presenting with cholestasis in our community (107/533), and could even be higher (28%) if Alagille syndrome, ciliopathies, and cystic fibrosis were included to the familial cholestasis group, and is the leading cause of need for liver transplantation in Saudi Arabia (15).

The ethnic background has been an important determinant of the spectrum of causes of cholestasis in different populations as some diseases prevail in certain racial and ethnic groups. For example, α1-AT deficiency is more common among Caucasians (up to 20%) (16), citrin deficiency has been found to be a common cause of IC among East Asians (5%) (17), and Niemann–Pick disease type C1 is important cause among the French Canadian of Acadian descent (18). In our population, the high frequency of neonatal-onset DJS (6%) is another feature that distinguishes IC in Saudi Arabia from other parts of the world except the Far-East Asia (Japan, Taiwan, Korea, and China) where the frequency of neonatal-onset DJS is in the range of 2% to 4.5% (19–21). The typical neonates with DJS are likely to be well looking with normal-ALT cholestasis, which resolves within 3 to 6 months of age (5). Early consideration and prompt diagnosis of DJS is a very important step in the work up of a neonate with cholestasis to avoid subjecting a patient with a benign prognosis to unnecessary invasive and costly evaluation. Another important genetic-based entity in our population is mitochondrial hepatopathy which contributed to 5.5% of the cases similar to DJS. The typical infants with mitochondrial hepatopathy manifest hypotonia, metabolic acidosis due to high serum lactate, and in some cases nystagmus (especially in *DGOUK* gene mutations) and abnormal findings on brain MRI (in about 64%) (3). It is important to make early diagnosis of this entity because mitochondrial hepatopathy is usually associated with systemic involvement which contraindicate liver transplantation. Another important observation in our data is absence of alpha-1 antitrypsin deficiency, similar to the data from East Asia, although we screen carefully for it during the diagnostic work up.

The old literature in the 1970s used the term “neonatal hepatitis” to describe unidentifiable causes of cholestasis (16, 22); INH comprised 40%–65% of IC in these days. Idiopathic NH was like a large black box and a puzzle for decades. However, during the past 2 decades, and because of the advancement in molecular and biochemical technologies, infants believed to have INH were later found to have different metabolic/genetic diseases; as a result, the frequency of INH reduced to 15%–20% in more recent studies (1). It is interesting that 30 years ago, in a single- center study of 64 Saudi infants with cholestasis, INH was “diagnosed” in 55% (23). Our present data showed that the addition of advanced molecular and biochemical testing to the armamentarium of the diagnostic process of IC has helped in decoding the puzzle of INH, and shrinking this black box from 55% to 30%. The proportion of INH could even be 10% if we included only the 415 of the 533 cases that were extensively investigated, including advanced molecular testing. With the easy availability of WES, and whole genome and RNA sequence in case of negative WES, the number of infants with identifiable mutations and genes resulting in cholestasis will continue to rise (24, 25).

As our hospital is a referral center for all regions in Saudi Arabia, as reflected from the regional distribution of the cases that we accepted over the study period, and due to the high prevalence of hereditary/genetic disorders within our population, we have been able through the application of various genetic studies to identify the genetic basis of many of the liver diseases causing IC (2, 5, 7, 26) and link some of these liver diseases to certain regions and tribes in Saudi Arabia (Figure 2). Our molecular analysis of 259 cases (49%) of IC revealed several variants unique to the Saudi population and are found in several major tribes in different regions of the Arabian Peninsula strongly suggesting that these variants are of common ancestral origin i.e., “founder gene mutations”, which accounted for the observed familial clusters seen in Figure 2. These findings have been very useful to us to focus our investigations and facilitate rapid molecular analysis by performing target gene analysis for the most common variants according to the region and the tribe.

The diagnostic challenge stems from the fact that IC is a common stereotypical manifestation of several perinatal insults to the highly vulnerable liver in young infants, with their limited capacity of bile acids metabolism, resulting in overlapping clinical, biochemical, and even histological responses. We believe that the knowledge on the epidemiology and spectrum of the causes of IC in the local population is an important first step in approaching cholestasis in any community and in determining the choice of laboratory tests in real clinical practice. Here, we outline a prioritized stepwise diagnostic algorithm that has been tailored to the local practice and authors experience. The approach recognizes recent advances in genetic technologies, important clinical and biochemical red flags, and the evolving field of precision medicine. In addition to implementing sophisticated laboratory investigations, our approach emphasizes on physicians to exercise clinical vigilance to detect subtle clinical signs and correlating them to facilitate the direction of specific biochemical tests and type of molecular analysis. Because the genetic/hereditary causes of IC contribute to 58% of IC in our population, our approach recognizes this feature and considers moving the genetic testing earlier in the diagnostic pathway after BA and other treatable disorders are evaluated and excluded in a timely manner. Careful selection of cases to undergo molecular analysis with the proper clinical context of consanguinity, similar family or tribal history, facial dysmorphism, systemic involvement, clinical and biochemical red flags suggestive of specific hereditary disease, or unidentified etiology despite extensive initial investigations in level 1 of assessment is expected to have high diagnostic yield on gene testing. Although not evaluated previously, such an approach of testing could be an efficient and cost-effective strategy.

The availability of the recent non-invasive diagnostic technologies has challenged the role of liver biopsy in the diagnostic work up of an infant with cholestasis, particularly if BA is not a clinical suspicion (27). In line of this approach, and consistent with the dramatic shift in the cholestasis evaluation paradigm, the number of liver biopsies in our center has reduced over time from 27/year in 2008 to 2–3/year in the last 3 years (Figure 3), when BA was highly suspected, as several pediatric liver diseases have become identifiable without the need for LB.

This is a retrospective single-center study with its inherent limitation of recall bias. There was a possibility of referral bias, therefore further studies are needed from other regions in Saudi Arabia to validate our results. Another limitation is the use of more than one gene panel from different laboratories during different times of the study period which might have affected the diagnostic yield of the algorithm.

In conclusion, in this report we showed what happened to INH in Saudi Arabia over the past 30 years and provided the missing information of the INH puzzle, which has a great impact on the differential diagnosis process, the diagnostic algorithm used, and the choice of laboratory tests in a clinical setting. Advancement in genomics has made the greatest impact in pediatric hepatology over the past two decades. Additional features of gene testing like rapid turnover and affordability at relatively low cost would secure a front place in the time-sensitive diagnostic algorithm of cholestasis.

## Data Availability

The raw data supporting the conclusions of this article will be made available by the authors, without undue reservation.
