# Supplementary material for: Patterns and unique features of infantile cholestasis among Arabs
Source: Front Pediatr. 2024 Jul 30;12:1423657. doi: 10.3389/fped.2024.1423657 (PMC11319143; doi:10.3389/fped.2024.1423657)
Supplement: Supplementary file 1 [file Datasheet1.pdf]

Cholestasis Panel in "Prevention Genetics Laboratory" Mayo Medical Lab

ABCB11, ABCB4, AKR1D1, ATP8B1, BAAT, CLDN1, HSD3B7, JAG1, NOTCH2, NR1H4, SERPINA1, SLC25A13, TJP2, VIPAS39, VPS33B

CGC Genetics panel

ABCB11, ABCB4, ABCC2, AKR1D1, AMACR, ATP8B1, BAAT, BCS1L, CC2D2A, CFTR, CLDN1, CYP27A1, CYP7A1, CYP7B1, DCDC2, DGUOK, GBA, HSD3B7, INVS, JAG1, LIPA, MKS1, MPV17, MYO5B, NOTCH2, NPC1, NPC2, NPHP3, NR1H4, PEX1, PEX2, PEX3, PEX5,PEX6, PEX7, PEX10, PEX11B, PEX12, PEX13, PEX14, PEX16, PEX19, PEX26, PKHD1, POLG1, POLG2, SERAC1, SERPINA1, SLC25A13, TJP2, TRMU, TTC37, VIPAS39, VPS33B
